# Dibromido(2,9-dimethyl-1,10-phenanthroline-κ^2^ *N*,*N*′)cobalt(II) acetonitrile monosolvate

**DOI:** 10.1107/S160053681204367X

**Published:** 2012-10-27

**Authors:** Sadif A. Shirvan, Manouchehr Aghajeri, Sara Haydari Dezfuli, Fereydoon Khazali, Ali Borsalani

**Affiliations:** a Department of Chemistry, Omidieh Branch, Islamic Azad University, Omidieh, Iran; b Department of Petroleum Engineering, Omidieh Branch, Islamic Azad University, Omidieh, Iran

## Abstract

In the title compound, [CoBr_2_(C_14_H_12_N_2_)]·CH_3_CN, the Co^II^ atom is four-coordinated in a distorted tetra­hedral geometry by two N atoms from a chelating 2,9-dimethyl-1,10-phenanthroline ligand and two terminal Br atoms. In the crystal, π–π contacts between the pyridine and benzene rings [centroid–centroid distances = 3.828 (5), 3.782 (5), 3.880 (5) and 3.646 (5) Å] stabilize the structure.

## Related literature

For related structures, see: Akbarzadeh Torbati *et al.* (2010 ▶); Alizadeh *et al.* (2009 ▶); Ding *et al.* (2006 ▶); Fanizzi *et al.* (1991 ▶); Lemoine *et al.* (2003 ▶); Robinson & Sinn (1975 ▶).
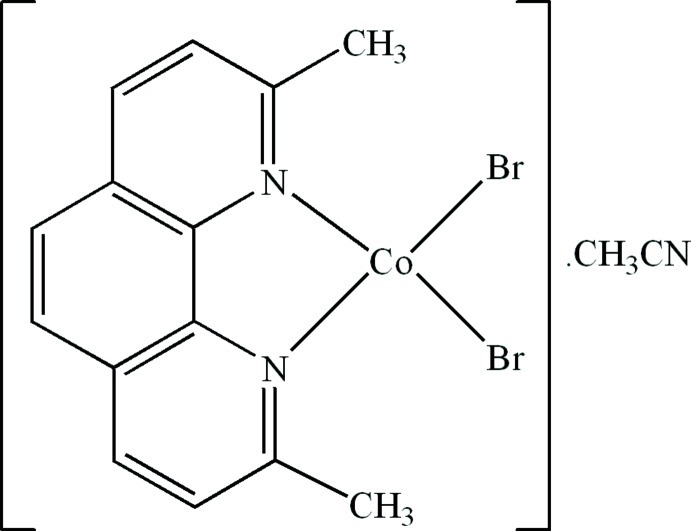


## Experimental

### 

#### Crystal data

[CoBr_2_(C_14_H_12_N_2_)]·C_2_H_3_N
*M* *_r_* = 468.04
Monoclinic, 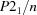

*a* = 7.6380 (5) Å
*b* = 12.7943 (6) Å
*c* = 17.9545 (11) Å
β = 101.128 (5)°
*V* = 1721.58 (18) Å^3^
*Z* = 4
Mo *K*α radiation
μ = 5.64 mm^−1^
*T* = 120 K
0.35 × 0.20 × 0.15 mm

#### Data collection

Bruker APEXII CCD diffractometer
Absorption correction: multi-scan (*SADABS*; Bruker, 2001 ▶) *T*_min_ = 0.259, *T*_max_ = 0.459
8417 measured reflections
3366 independent reflections
2345 reflections with *I* > 2σ(*I*)
*R*_int_ = 0.101

#### Refinement

*R*[*F* ^2^ > 2σ(*F* ^2^)] = 0.075
*wR*(*F* ^2^) = 0.194
*S* = 1.02
3366 reflections
200 parameters
H-atom parameters constrained
Δρ_max_ = 1.11 e Å^−3^
Δρ_min_ = −1.03 e Å^−3^

### 

Data collection: *APEX2* (Bruker, 2007 ▶); cell refinement: *SAINT* (Bruker, 2007 ▶); data reduction: *SAINT*; program(s) used to solve structure: *SHELXS97* (Sheldrick, 2008 ▶); program(s) used to refine structure: *SHELXL97* (Sheldrick, 2008 ▶); molecular graphics: *ORTEP-3* (Farrugia, 1997 ▶) and *Mercury* (Macrae *et al.*, 2006 ▶); software used to prepare material for publication: *SHELXTL* (Sheldrick, 2008 ▶).

## Supplementary Material

Crystal structure: contains datablock(s) I, global. DOI: 10.1107/S160053681204367X/hy2596sup1.cif

Structure factors: contains datablock(s) I. DOI: 10.1107/S160053681204367X/hy2596Isup2.hkl

Additional supplementary materials:  crystallographic information; 3D view; checkCIF report

## Figures and Tables

**Table 1:** Selected bond lengths (Å)

Co1—N1	2.051 (8)
Co1—N2	2.036 (8)
Co1—Br1	2.3592 (14)
Co1—Br2	2.3682 (14)

**Fig. 1.**
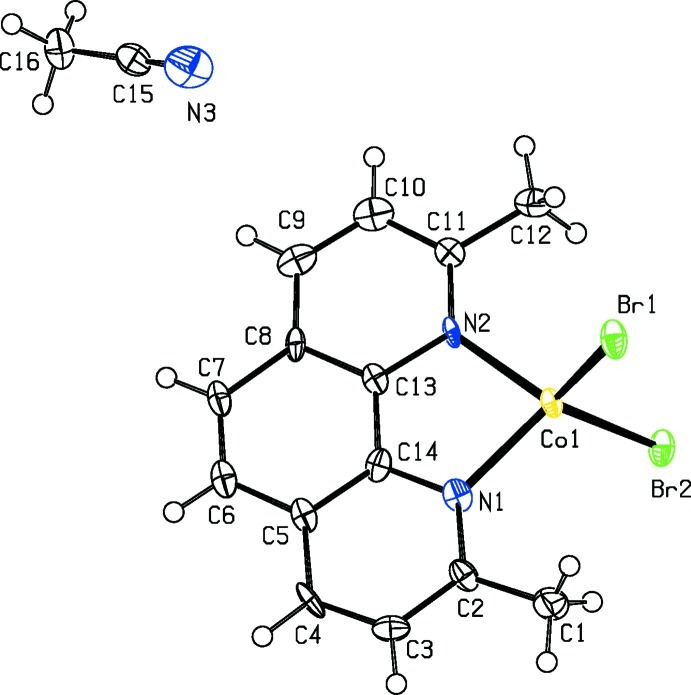
The molecular structure of the title compound. Displacement ellipsoids are drawn at the 50% probability level.

**Fig. 2.**
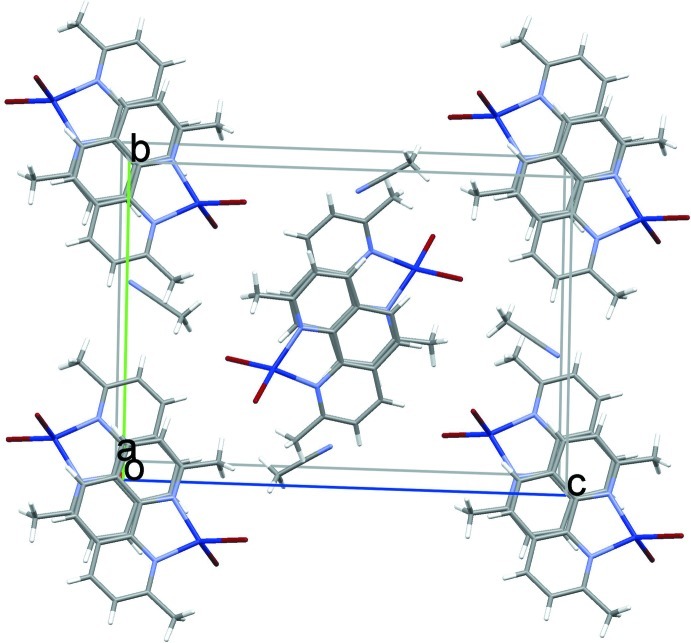
Crystal packing diagram for the title compound.

## supplementary crystallographic information

### Comment

2,9-Dimethyl-1,10-phenanthroline (dmphen) is a good bidentate ligand, and numerous complexes with dmphen have been prepared, such as that of mercury (Alizadeh *et al.*, 2009), copper (Lemoine *et al.*, 2003), nickel (Ding *et al.*, 2006), gold (Robinson & Sinn, 1975), platinum (Fanizzi *et al.*, 1991) and cobalt (Akbarzadeh Torbati *et al.*, 2010). Here, we report the synthesis and structure of the title compound.

In the title compound (Fig. 1), the Co^II^ atom is four-coordinated in a distorted tetrahedral geometry by two N atoms from a chelating dmphen ligand and two terminal Br atoms. The Co—Br and Co—N bond lengths (Table 1) and angles are normal. In the crystal, π–π contacts between the pyridine and benzene rings (Fig. 2), *Cg*3···*Cg*3^i^, *Cg*3···*Cg*4^i^, *Cg*3···*Cg*4^ii^ and *Cg*4···*Cg*4^ii^ [symmetry codes: (i) -x, 1-y, 1-z; (ii) 1-x, 1-y, 1-z, *Cg*3 and *Cg*4 are the centroids of the N2, C8–C11, C13 ring and C5–C8, C13, C14 ring, respectively], with centroid–centroid distances of 3.828 (5), 3.782 (5), 3.880 (5) and 3.646 (5) Å, stabilize the structure.

### Experimental

For the preparation of the title compound, a solution of 2,9-dimethyl-1,10-phenanthroline (0.28 g, 1.33 mmol) in methanol (20 ml) was added to a solution of CoBr_2_ (0.29 g, 1.33 mmol) in acetonitrile (15 ml) and the resulting blue solution was stirred for 15 min at 313 K. This solution was left to evaporate slowly at room temperature. After one week, blue block crystals of the title compound were isolated (yield: 0.46 g, 73.9%).

### Refinement

All H atoms were positioned geometrically and refined as riding atoms, with C—H = 0.93 (aromatic) and 0.96 (CH_3_) Å and with *U*_iso_(H) = 1.2(1.5 for methyl)*U*_eq_(C). The highest residual electron density was found 0.92 Å from Br2 the deepest hole 1.02 Å from Br1.

### Crystal data

**Table:**

[CoBr_2_(C_14_H_12_N_2_)]·C_2_H_3_N	*F*(000) = 916
*M**_r_* = 468.04	*D*_x_ = 1.806 Mg m^−^^3^
Monoclinic, *P*2_1_/*n*	Mo *K*α radiation, λ = 0.71073 Å
Hall symbol: -P 2yn	Cell parameters from 8417 reflections
*a* = 7.6380 (5) Å	θ = 2.0–26.0°
*b* = 12.7943 (6) Å	µ = 5.64 mm^−^^1^
*c* = 17.9545 (11) Å	*T* = 120 K
β = 101.128 (5)°	Block, blue
*V* = 1721.58 (18) Å^3^	0.35 × 0.20 × 0.15 mm
*Z* = 4	

### Data collection

**Table:**

Bruker APEXII CCD diffractometer	3366 independent reflections
Radiation source: fine-focus sealed tube	2345 reflections with *I* > 2σ(*I*)
Graphite monochromator	*R*_int_ = 0.101
φ and ω scans	θ_max_ = 26.0°, θ_min_ = 2.0°
Absorption correction: multi-scan (*SADABS*; Bruker, 2001)	*h* = −9→9
*T*_min_ = 0.259, *T*_max_ = 0.459	*k* = −15→13
8417 measured reflections	*l* = −22→20

### Refinement

**Table:**

Refinement on *F*^2^	Primary atom site location: structure-invariant direct methods
Least-squares matrix: full	Secondary atom site location: difference Fourier map
*R*[*F*^2^ > 2σ(*F*^2^)] = 0.075	Hydrogen site location: inferred from neighbouring sites
*wR*(*F*^2^) = 0.194	H-atom parameters constrained
*S* = 1.02	*w* = 1/[σ^2^(*F*_o_^2^) + (0.1187*P*)^2^] where *P* = (*F*_o_^2^ + 2*F*_c_^2^)/3
3366 reflections	(Δ/σ)_max_ = 0.003
200 parameters	Δρ_max_ = 1.11 e Å^−^^3^
0 restraints	Δρ_min_ = −1.03 e Å^−^^3^

### Special details

**Table:**

Geometry. All e.s.d.'s (except the e.s.d. in the dihedral angle between two l.s. planes) are estimated using the full covariance matrix. The cell e.s.d.'s are taken into account individually in the estimation of e.s.d.'s in distances, angles and torsion angles; correlations between e.s.d.'s in cell parameters are only used when they are defined by crystal symmetry. An approximate (isotropic) treatment of cell e.s.d.'s is used for estimating e.s.d.'s involving l.s. planes.
Refinement. Refinement of *F*^2^ against ALL reflections. The weighted *R*-factor *wR* and goodness of fit *S* are based on *F*^2^, conventional *R*-factors *R* are based on *F*, with *F* set to zero for negative *F*^2^. The threshold expression of *F*^2^ > σ(*F*^2^) is used only for calculating *R*-factors(gt) *etc*. and is not relevant to the choice of reflections for refinement. *R*-factors based on *F*^2^ are statistically about twice as large as those based on *F*, and *R*- factors based on ALL data will be even larger.

### Fractional atomic coordinates and isotropic or equivalent isotropic displacement parameters (Å^2^)

**Table:**

	*x*	*y*	*z*	*U*_iso_*/*U*_eq_	
Co1	0.15502 (14)	0.35537 (10)	0.33642 (7)	0.0162 (3)	
Br1	0.31107 (12)	0.37421 (8)	0.23591 (6)	0.0249 (3)	
Br2	−0.12001 (11)	0.26909 (8)	0.29292 (6)	0.0233 (3)	
C1	0.3312 (15)	0.1227 (8)	0.3918 (6)	0.032 (2)	
H1A	0.3906	0.1476	0.3528	0.038*	
H1B	0.2067	0.1129	0.3711	0.038*	
H1C	0.3828	0.0574	0.4112	0.038*	
C2	0.3520 (11)	0.2011 (8)	0.4548 (5)	0.020 (2)	
C3	0.4380 (12)	0.1773 (8)	0.5287 (6)	0.024 (2)	
H3	0.4825	0.1103	0.5397	0.029*	
C4	0.4587 (10)	0.2517 (8)	0.5861 (5)	0.022 (2)	
H4	0.5151	0.2350	0.6353	0.026*	
C5	0.3920 (10)	0.3531 (8)	0.5678 (5)	0.0176 (19)	
C6	0.4112 (11)	0.4362 (8)	0.6219 (6)	0.022 (2)	
H6	0.4712	0.4237	0.6712	0.026*	
C7	0.3452 (11)	0.5316 (8)	0.6035 (5)	0.022 (2)	
H7	0.3570	0.5835	0.6404	0.026*	
C8	0.2557 (10)	0.5549 (7)	0.5268 (5)	0.0178 (19)	
C9	0.1829 (12)	0.6513 (8)	0.5024 (7)	0.026 (2)	
H9	0.1894	0.7065	0.5365	0.031*	
C10	0.1008 (12)	0.6654 (8)	0.4275 (7)	0.028 (2)	
H10	0.0564	0.7308	0.4107	0.033*	
C11	0.0845 (10)	0.5820 (7)	0.3772 (5)	0.0166 (18)	
C12	−0.0028 (13)	0.5960 (8)	0.2951 (6)	0.027 (2)	
H12A	−0.1007	0.5481	0.2824	0.033*	
H12B	0.0828	0.5825	0.2635	0.033*	
H12C	−0.0460	0.6664	0.2870	0.033*	
C13	0.2355 (10)	0.4741 (7)	0.4725 (5)	0.0165 (19)	
C14	0.3071 (10)	0.3727 (7)	0.4935 (6)	0.0182 (19)	
C15	0.1720 (12)	0.9623 (8)	0.5702 (6)	0.027 (2)	
C16	0.1350 (16)	1.0197 (10)	0.6364 (7)	0.039 (3)	
H16A	0.2341	1.0645	0.6560	0.059*	
H16B	0.1176	0.9710	0.6750	0.059*	
H16C	0.0293	1.0612	0.6216	0.059*	
N1	0.2885 (9)	0.2967 (6)	0.4379 (4)	0.0182 (16)	
N2	0.1506 (8)	0.4887 (6)	0.3979 (4)	0.0138 (15)	
N3	0.2013 (13)	0.9203 (8)	0.5206 (7)	0.043 (3)	

### Atomic displacement parameters (Å^2^)

**Table:**

	*U*^11^	*U*^22^	*U*^33^	*U*^12^	*U*^13^	*U*^23^
Co1	0.0134 (5)	0.0219 (7)	0.0121 (6)	−0.0001 (5)	−0.0010 (4)	−0.0009 (5)
Br1	0.0201 (4)	0.0384 (6)	0.0167 (5)	−0.0082 (4)	0.0050 (4)	−0.0041 (4)
Br2	0.0160 (4)	0.0306 (6)	0.0222 (5)	−0.0061 (4)	0.0014 (3)	−0.0057 (4)
C1	0.042 (6)	0.025 (6)	0.027 (6)	0.011 (5)	0.005 (5)	0.000 (5)
C2	0.016 (4)	0.028 (5)	0.017 (5)	0.007 (4)	0.002 (3)	0.002 (4)
C3	0.022 (4)	0.018 (5)	0.032 (6)	0.005 (4)	0.008 (4)	0.006 (4)
C4	0.011 (4)	0.041 (6)	0.011 (5)	0.006 (4)	−0.004 (3)	0.008 (4)
C5	0.006 (3)	0.032 (5)	0.015 (5)	−0.001 (3)	0.003 (3)	0.000 (4)
C6	0.013 (4)	0.037 (6)	0.015 (5)	−0.007 (4)	0.004 (3)	−0.002 (4)
C7	0.019 (4)	0.033 (6)	0.012 (5)	−0.005 (4)	0.002 (4)	−0.002 (4)
C8	0.011 (4)	0.026 (5)	0.015 (5)	−0.006 (3)	−0.001 (3)	−0.006 (4)
C9	0.016 (4)	0.022 (5)	0.041 (7)	−0.002 (4)	0.013 (4)	0.000 (5)
C10	0.018 (4)	0.027 (6)	0.040 (7)	0.003 (4)	0.010 (4)	0.003 (5)
C11	0.009 (4)	0.023 (5)	0.019 (5)	0.000 (3)	0.007 (3)	0.002 (4)
C12	0.025 (5)	0.024 (5)	0.034 (6)	0.000 (4)	0.006 (4)	0.008 (5)
C13	0.007 (4)	0.027 (5)	0.015 (5)	−0.005 (3)	0.003 (3)	0.002 (4)
C14	0.008 (4)	0.024 (5)	0.023 (5)	−0.004 (3)	0.002 (3)	−0.005 (4)
C15	0.023 (4)	0.030 (6)	0.027 (6)	0.005 (4)	0.002 (4)	0.005 (5)
C16	0.045 (6)	0.041 (7)	0.026 (6)	−0.007 (5)	−0.005 (5)	−0.003 (5)
N1	0.008 (3)	0.027 (4)	0.020 (4)	0.007 (3)	0.004 (3)	−0.001 (3)
N2	0.006 (3)	0.025 (4)	0.009 (4)	−0.003 (3)	−0.003 (3)	−0.001 (3)
N3	0.043 (5)	0.042 (6)	0.050 (7)	0.019 (5)	0.018 (5)	0.001 (5)

### Geometric parameters (Å, º)

**Table:**

Co1—N1	2.051 (8)	C8—C9	1.390 (14)
Co1—N2	2.036 (8)	C8—C13	1.409 (13)
Co1—Br1	2.3592 (14)	C9—C10	1.382 (16)
Co1—Br2	2.3682 (14)	C9—H9	0.9300
C1—C2	1.497 (14)	C10—C11	1.387 (14)
C1—H1A	0.9600	C10—H10	0.9300
C1—H1B	0.9600	C11—N2	1.321 (12)
C1—H1C	0.9600	C11—C12	1.507 (14)
C2—N1	1.329 (12)	C12—H12A	0.9600
C2—C3	1.395 (14)	C12—H12B	0.9600
C3—C4	1.389 (14)	C12—H12C	0.9600
C3—H3	0.9300	C13—N2	1.383 (11)
C4—C5	1.409 (14)	C13—C14	1.429 (13)
C4—H4	0.9300	C14—N1	1.382 (12)
C5—C14	1.389 (13)	C15—N3	1.099 (15)
C5—C6	1.428 (14)	C15—C16	1.471 (16)
C6—C7	1.337 (14)	C16—H16A	0.9600
C6—H6	0.9300	C16—H16B	0.9600
C7—C8	1.444 (13)	C16—H16C	0.9600
C7—H7	0.9300		
			
N2—Co1—N1	83.3 (3)	C10—C9—H9	119.9
N2—Co1—Br1	113.1 (2)	C8—C9—H9	119.9
N1—Co1—Br1	118.62 (19)	C9—C10—C11	119.9 (9)
N2—Co1—Br2	117.58 (18)	C9—C10—H10	120.0
N1—Co1—Br2	112.3 (2)	C11—C10—H10	120.0
Br1—Co1—Br2	110.02 (6)	N2—C11—C10	122.1 (9)
C2—C1—H1A	109.5	N2—C11—C12	117.1 (8)
C2—C1—H1B	109.5	C10—C11—C12	120.8 (9)
H1A—C1—H1B	109.5	C11—C12—H12A	109.5
C2—C1—H1C	109.5	C11—C12—H12B	109.5
H1A—C1—H1C	109.5	H12A—C12—H12B	109.5
H1B—C1—H1C	109.5	C11—C12—H12C	109.5
N1—C2—C3	120.1 (9)	H12A—C12—H12C	109.5
N1—C2—C1	117.5 (8)	H12B—C12—H12C	109.5
C3—C2—C1	122.3 (9)	N2—C13—C8	122.6 (8)
C4—C3—C2	121.4 (9)	N2—C13—C14	117.6 (8)
C4—C3—H3	119.3	C8—C13—C14	119.8 (8)
C2—C3—H3	119.3	N1—C14—C5	122.0 (8)
C3—C4—C5	118.2 (9)	N1—C14—C13	117.8 (8)
C3—C4—H4	120.9	C5—C14—C13	120.1 (8)
C5—C4—H4	120.9	N3—C15—C16	179.1 (13)
C14—C5—C4	118.1 (9)	C15—C16—H16A	109.5
C14—C5—C6	119.0 (9)	C15—C16—H16B	109.5
C4—C5—C6	122.9 (9)	H16A—C16—H16B	109.5
C7—C6—C5	121.9 (9)	C15—C16—H16C	109.5
C7—C6—H6	119.1	H16A—C16—H16C	109.5
C5—C6—H6	119.1	H16B—C16—H16C	109.5
C6—C7—C8	120.7 (9)	C2—N1—C14	120.0 (8)
C6—C7—H7	119.7	C2—N1—Co1	129.6 (7)
C8—C7—H7	119.7	C14—N1—Co1	110.4 (6)
C9—C8—C13	116.7 (9)	C11—N2—C13	118.5 (8)
C9—C8—C7	124.8 (9)	C11—N2—Co1	130.6 (6)
C13—C8—C7	118.5 (9)	C13—N2—Co1	110.9 (6)
C10—C9—C8	120.1 (10)		
			
N1—C2—C3—C4	0.4 (13)	C1—C2—N1—C14	−178.8 (8)
C1—C2—C3—C4	178.9 (9)	C3—C2—N1—Co1	−179.3 (6)
C2—C3—C4—C5	−0.7 (13)	C1—C2—N1—Co1	2.1 (12)
C3—C4—C5—C14	0.8 (11)	C5—C14—N1—C2	0.4 (12)
C3—C4—C5—C6	−177.9 (8)	C13—C14—N1—C2	−179.6 (7)
C14—C5—C6—C7	1.9 (12)	C5—C14—N1—Co1	179.6 (6)
C4—C5—C6—C7	−179.3 (8)	C13—C14—N1—Co1	−0.4 (8)
C5—C6—C7—C8	−1.8 (12)	N2—Co1—N1—C2	179.2 (7)
C6—C7—C8—C9	179.9 (8)	Br1—Co1—N1—C2	−68.1 (8)
C6—C7—C8—C13	1.6 (12)	Br2—Co1—N1—C2	62.1 (7)
C13—C8—C9—C10	−2.0 (12)	N2—Co1—N1—C14	0.0 (5)
C7—C8—C9—C10	179.6 (8)	Br1—Co1—N1—C14	112.7 (5)
C8—C9—C10—C11	2.6 (13)	Br2—Co1—N1—C14	−117.1 (5)
C9—C10—C11—N2	−2.2 (13)	C10—C11—N2—C13	1.2 (11)
C9—C10—C11—C12	−179.3 (8)	C12—C11—N2—C13	178.4 (7)
C9—C8—C13—N2	1.1 (11)	C10—C11—N2—Co1	−177.4 (6)
C7—C8—C13—N2	179.5 (7)	C12—C11—N2—Co1	−0.2 (10)
C9—C8—C13—C14	180.0 (7)	C8—C13—N2—C11	−0.7 (11)
C7—C8—C13—C14	−1.6 (11)	C14—C13—N2—C11	−179.6 (7)
C4—C5—C14—N1	−0.7 (11)	C8—C13—N2—Co1	178.2 (6)
C6—C5—C14—N1	178.1 (7)	C14—C13—N2—Co1	−0.7 (8)
C4—C5—C14—C13	179.3 (7)	N1—Co1—N2—C11	179.1 (7)
C6—C5—C14—C13	−1.9 (11)	Br1—Co1—N2—C11	60.8 (7)
N2—C13—C14—N1	0.7 (10)	Br2—Co1—N2—C11	−69.2 (7)
C8—C13—C14—N1	−178.2 (7)	N1—Co1—N2—C13	0.4 (5)
N2—C13—C14—C5	−179.3 (7)	Br1—Co1—N2—C13	−117.9 (5)
C8—C13—C14—C5	1.8 (11)	Br2—Co1—N2—C13	112.1 (5)
C3—C2—N1—C14	−0.2 (12)		
